# Elastic modulus-reflected liver lesion stiffness relates to worse prognosis in pancreatic cancer patients with liver metastasis

**DOI:** 10.1186/s12957-023-03140-4

**Published:** 2023-08-24

**Authors:** Shu Dong, Xian Miao, Ke Zhang, Xiaoyan Zhu, Yi Gao, Hao Chen

**Affiliations:** 1 Department of Integrative Oncology, Fudan University Shanghai Cancer Center, Shanghai, China; 2 Department of Oncology, Shanghai Medical College, Fudan University, Shanghai, 200032 China; 3 Department of Oncology, Nantong Hospital of Traditional Chinese Medicine, Nantong, 226001 Jiangsu Province China; 4 Department of Medical Ultrasound, Fudan University Shanghai Cancer Center, Shanghai, 200032 China

**Keywords:** Pancreatic cancer, Liver metastasis, Elastic modulus, Stiffness, Prognosis

## Abstract

**Background:**

Liver stiffness relates to more advanced tumor status and poor outcomes in primary liver cancer, while its prognostic role in pancreatic cancer with liver metastasis is unclear. Therefore, the current study aimed to explore the correlation of elastic modulus (EM)-reflected liver lesion stiffness with clinical characteristics, tumor markers, and survival among pancreatic cancer patients with liver metastasis.

**Methods:**

Fifty-four pancreatic cancer patients with liver metastasis were enrolled, and the EM of liver metastasis and peripheral liver tissue was measured by two-dimensional shear wave elastography. Relative EM was calculated as the ratio of EM in liver metastasis to that in peripheral liver tissue, which reflected the relative liver lesion stiffness.

**Results:**

The median relative EM of liver metastasis was 7.8 (interquartile range: 4.8–10.7) folds. Relative EM of liver metastasis was correlated with primary pancreatic cancer location (*P* = 0.048), the presence of extra lung metastasis (*P* = 0.040), liver metastasis ≥ 3 cm (*P* = 0.007), and the absence of extraskeletal metastasis (*P* = 0.036); but it was not correlated with tumor markers such as CA199, CA125, or CEA (all *P* > 0.05). Encouragingly, high relative EM of liver metastasis (cut off by median value) was correlated with poor progression-free survival (PFS) (*P* = 0.032) but not overall survival (OS) (*P* = 0.285). Multivariable Cox analysis showed that high relative EM of liver metastasis (hazard ratio (HR) = 1.768, *P* = 0.048) and multiple metastases (HR = 2.262, *P* = 0.036) independently predicted decreased PFS, but only abnormal CEA independently forecasted decreased OS (HR = 2.390, *P* = 0.027).

**Conclusion:**

Elastic modulus reflected liver lesion stiffness may predict a worse prognosis in pancreatic cancer patients with liver metastasis.

**Supplementary Information:**

The online version contains supplementary material available at 10.1186/s12957-023-03140-4.

## Introduction

Pancreatic cancer is a common cancer with high mortality globally and is projected to be the second-highest cause of cancer-related death in the United States by 2030 [1–3]. Currently, surgical resection followed by adjuvant chemotherapy provides relatively acceptable outcomes among early-stage pancreatic cancer patients [4, 5]. Unfortunately, a large proportion of pancreatic cancer patients are diagnosed at an advanced stage with distant metastasis [6]. The liver is one of the most common metastatic sites in advanced pancreatic cancer patients, and patients with liver metastasis are not eligible for surgery; their prognosis is extremely dismal, with a 5-year overall survival (OS) rate of approximately 2% [7, 8]. Therefore, exploring potential indicators to reflect prognosis is crucial to improving the management of these patients.

Liver stiffness is able to reflect the degree of liver fibrosis, whose measurement plays an essential clinical role in reflecting the disease risk and prognosis of liver cancer [9–13]. For instance, the liver stiffness parameter is able to predict the occurrence of liver cancer among hepatitis C virus patients [11]. Elevated liver stiffness is correlated with deteriorated liver injury and decreased OS in advanced liver cancer patients [14]. Moreover, early-stage liver cancer patients who have increased liver stiffness after surgery face a higher risk of relapse [15]. In addition, liver stiffness can be used to monitor treatment response among colorectal cancer patients with liver metastasis undergoing transarterial chemoembolization [16]. In the preclinical setting, it is reported that low stiffness of liver metastasis is associated with survival in mice with pancreatic cancer liver metastasis [17]. Moreover, the stiffness of pancreatic cancer is associated with tumor size in mice [18]. Taken together, we deduced that liver stiffness might also play a crucial role in pancreatic cancer patients with liver metastasis, while related information is scarce.

Thus, the current study aimed to explore the correlation of liver stiffness (measured via relative elastic modulus (EM)) with clinical characteristics, tumor markers, and survival among pancreatic cancer patients with liver metastasis.

## Methods

### Patients

Between March 2017 and April 2020, 54 pancreatic cancer patients with liver metastasis were serially included in this study. The inclusion criteria were set as follows: (i) diagnosed with primary pancreatic cancer; (ii) confirmed as liver metastasis by image logical examination, such as contrast-enhanced computerized tomography (CT); and (iii) age over 18 years. The exclusion criteria were as follows: (i) with other primary cancers or hematologic malignancies; (ii) with fatty liver, cirrhosis, or other liver diseases; and (iii) pregnant or lactating female patients. This study was approved by the Ethics Committee of Fudan University Shanghai Cancer Center.

### Documents

Clinical features of pancreatic cancer patients were obtained after enrollment, including age, sex, tumor number, number of liver metastases, size of liver metastasis, extrahepatic metastasis, tumor markers, and treatment information. Patients were followed up regularly until April 2021. The median follow-up period was 10 months with a range of 3–26 months. Then, progression-free survival (PFS) and overall survival (OS) were imputed.

### Evaluation

Liver stiffness of liver metastasis and peripheral liver tissue was measured using two-dimensional shear wave elastography (2D-SWE) by an Aixplorer US imaging system (SuperSonic Imagine, France) with an SC6–1 (frequency of 1–6 MHz) convex probe. For patients with multiple liver metastases, liver stiffness was measured based on tumors 2–5 cm in diameter and without blood vessels or biliary ducts around the tumor. The procedure was the same as in a previous study and was in line with the European Federation of Societies for Ultrasound in Medicine and Biology (EFSUMB) guidelines [19, 20]. In brief, the probe was positioned into the patients’ intercostal spaces of the right lobe of the liver. The 2D-SWE was carried out followed by a real-time B-mode ultrasound scan to ensure the target area of the liver. The size of the sampling box was 4 cm × 3 cm, and the top edge of the box was located approximately 1 cm under Glisson’s capsule of the liver. Patients were asked to hold their breath for approximately 5 s. Then, the images were stabilized, and the elastic modulus (EM) was automatically measured and displayed (a representative image is shown in Fig. S1). Each patient received three times of measurements, and the mean value was calculated based on the three measurements. The measurement of the stiffness of liver metastasis and peripheral liver tissue was conducted by two sonographers who had ultrasound professional qualification certificates and experience in sonography for over 10 years. The EM value was calculated as the mean values of the EM values measured by the two sonographers. After liver stiffness measurement, relative EM was calculated, defined as the ratio of EM in liver metastasis to EM in peripheral liver tissue. The median relative EM was used to classify patients into high relative EM and low relative EM groups.

### Statistics

SPSS 26.0 (IBM Corp., USA) and GraphPad Prism 7.01 (GraphPad Software Inc., USA) was employed for data analysis and graph construction, respectively. Data are displayed as the mean ± standard deviation (SD) for normally distributed continuous variables, median (interquartile range (IQR)) for skewed-distributed continuous variables, and count (percentage) for categorized variables. The correlation of EM with clinical characteristics was determined using the Wilcoxon rank sum test. PFS and OS were plotted using Kaplan–Meier (KM) curves and determined by the Breslow test. Independent prognostic factors were analyzed using multivariable Cox proportional hazard regression analysis with the backward stepwise method, and all factors were included in the analysis. A *P* value < 0.05 was considered significant.

## Results

### Clinical features

The mean age of the 54 included patients was 62.0 ± 9.7 years. Meanwhile, there were 29 (53.7%) males and 25 (46.3%) females. Regarding tumor location, 17 (31.5%) patients’ tumors were located at the head of the pancreas, 17 (31.5%) patients’ tumors were located at the body of the pancreas, and 29 (53.7%) patients’ tumors were located at the cauda of the pancreas (there were 9 patients with two lesions in the pancreas). Nine (16.7%) patients had a single metastasis in the liver and 45 (83.3%) patients had multiple metastases in the liver. The median (IQR) liver metastasis was 2.7 (1.5–3.5) cm. For tumor markers, the median (IQR) values of carbohydrate antigen 199 (CA199), cancer antigen 125 (CA125), and carcinoembryonic antigen (CEA) were 1000.0 (59.7–1000.0) U/mL, 78.2 (33.3–227.9) U/mL, and 14.4 (4.9–67.0) ng/mL, respectively. In terms of liver stiffness, the median (IQR) values of EM of liver metastasis, EM of peripheral liver tissue, and relative EM were 57.9 (43.3–83.5), 7.5 (6.3–10.4), and 7.8 (4.8–10.7), respectively (Table 1).

**Table 1:** Clinical features

Items	Patients (*N* = 54)
Age (years), mean ± SD	62.0 ± 9.7
Gender, no. (%)	
Male	29 (53.7)
Female	25 (46.3)
Tumor location, no. (%)	
Head of pancreas	17 (31.5)
Body of pancreas	17 (31.5)
Cauda of pancreas	29 (53.7)
Liver metastasis, no. (%)	54 (100.0)
Single	9 (16.7)
Multiple	45 (83.3)
Extrahepatic metastasis, no. (%)	
Intra-abdomen	34 (63.0)
Lymph gland	4 (7.4)
Skeleton	6 (11.1)
Lung	3 (5.6)
No extrahepatic metastases	12 (22.2)
Number of extrahepatic metastases, no. (%)	
0	12 (22.2)
1	18 (33.3)
2	14 (25.9)
3	8 (14.8)
4	1 (1.9)
5	1 (1.9)
Size of liver metastasis (cm), median (IQR)	2.7 (1.5–3.5)
Tumor markers, median (IQR)	
CA199 (U/mL)	1000.0 (59.7–1000.0)
CA125 (U/mL)	78.2 (33.3–227.9)
CEA (ng/mL)	14.4 (4.9–67.0)
Treatment, No. (%)	
AG	33 (61.1)
Other GEM-based regimens	33 (61.1)
Folfirinox	5 (9.3)
Others	14 (25.9)
Liver stiffness, median (IQR)	
EM of liver metastasis	57.9 (43.3–83.5)
EM of peripheral liver tissue	7.5 (6.3–10.4)
Relative EM	7.8 (4.8–10.7)

*SD* Standard deviation, *IQR* Interquartile range, *CA199* Carbohydrate antigen 199, *CA125* Cancer antigen 125, *CEA* Carcinoembryonic antigen, *AG* Albumin paclitaxel + gemcitabine, *GEM* Gemcitabine, *EM* Elastic modulus

### Correlation between relative EM and clinical characteristics

Elevated relative EM was correlated with tumors location in the cauda of the pancreas (*P* = 0.048), lung metastasis (*P* = 0.040), and liver metastasis ≥ 3 cm (*P* = 0.007), while decreased relative EM was correlated with skeletal metastasis (*P* = 0.036). Moreover, no correlation was found in relative EM with other clinical characteristics (all *P* > 0.05) (Table 2).

**Table 2:** Correlation of relative EM with clinical characteristics

Items	Relative EM, median (IQR)	*Z* value	*P* value
Age		− 0.976	0.329
< 60 years	7.7 (4.4–9.7)		
≥ 60 years	7.8 (4.9–13.1)		
Gender		− 0.842	0.400
Male	6.8 (4.7–10.3)		
Female	8.8 (4.7–13.4)		
Live metastases		− 1.521	0.128
Single	10.1 (6.3–13.4)		
Multiple	6.8 (4.5–10.3)		
Head of pancreas		− 1.584	0.113
No	8.8 (5.0–11.0)		
Yes	5.5 (4.0–9.8)		
Body of pancreas		− 0.596	0.551
No	7.7 (4.4–11.0)		
Yes	7.8 (5.2–10.4)		
Cauda of pancreas		− 1.978	**0.048**
No	5.7 (4.3–9.5)		
Yes	9.3 (5.1–13.1)		
Number of extrahepatic metastases		− 0.618	0.536
0–1	8.1 (5.1–10.3)		
> 1	5.6 (4.3–11.1)		
Enterocoelia metastasis		− 0.502	0.616
No	7.9 (4.3–10.6)		
Yes	7.8 (5.0–10.8)		
Lymph gland metastasis		− 1.883	0.060
No	8.1 (4.9–10.9)		
Yes	4.5 (2.7–7.3)		
Skeleton metastasis		− 2. 092	**0.036**
No	8.6 (4.9–11.1)		
Yes	4.7 (3.4–7.1)		
Lung metastasis		− 2.059	**0.040**
No	7.7 (4.5–10.6)		
Yes	14.0 (10.1–NR)		
No extrahepatic metastases		− 0.812	0.417
No	7.3 (4.5–10.6)		
Yes	9.0 (5.0–12.8)		
Size of liver metastasis		− 2.707	**0.007**
< 3 cm	5.2 (4.3–9.9)		
≥ 3 cm	9.3 (7.2–11.6)		
AG		− 0.479	0.632
No	8.8 (5.3–10.4)		
Yes	5.7 (4.4–11.9)		
Other GEM-based regimens		− 0.275	0.783
No	8.0 (4.7–10.3)		
Yes	7.8 (4.7–11.6)		
Folfirinox		− 0.463	0.644
No	7.8 (4.9–10.7)		
Yes	8.8 (2.4–12.9)		
Others		− 0.573	0.567
No	7.8 (4.6–10.5)		
Yes	7.2 (4.8–14.1)		

*EM* Elastic modulus, *IQR* Interquartile range, *NR* Not reported, *GEM* Gemcitabine

### Correlation between relative EM and tumor markers

To further explore the association of relative EM with tumor markers, the relative EM was investigated in patients with abnormal as well as normal CA199, CA125, and CEA. However, no correlation was found in relative EM with these tumor markers (all *P* > 0.05) (Table 3).

**Table 3:** Correlation of relative EM with tumor markers

Items	Relative EM, median (IQR)	*Z* value	*P* value
CA199		− 0.666	0.505
Abnormal	5.7 (4.4–8.8)		
Normal	8.3 (4.9–10.8)		
CA125		− 0.473	0.636
Abnormal	9.7 (4.4–13.3)		
Normal	7.7 (4.9–10.1)		
CEA		− 0.688	0.491
Abnormal	9.7 (5.2–11.6)		
Normal	7.8 (4.5–10.3)		

*EM* Elastic modulus, *IQR* Interquartile range, *CA199* Carbohydrate antigen 199, *CA125* Cancer antigen 125, *CEA* Carcinoembryonic antigen

### Correlation of relative EM with PFS and OS

**Fig. 1:**
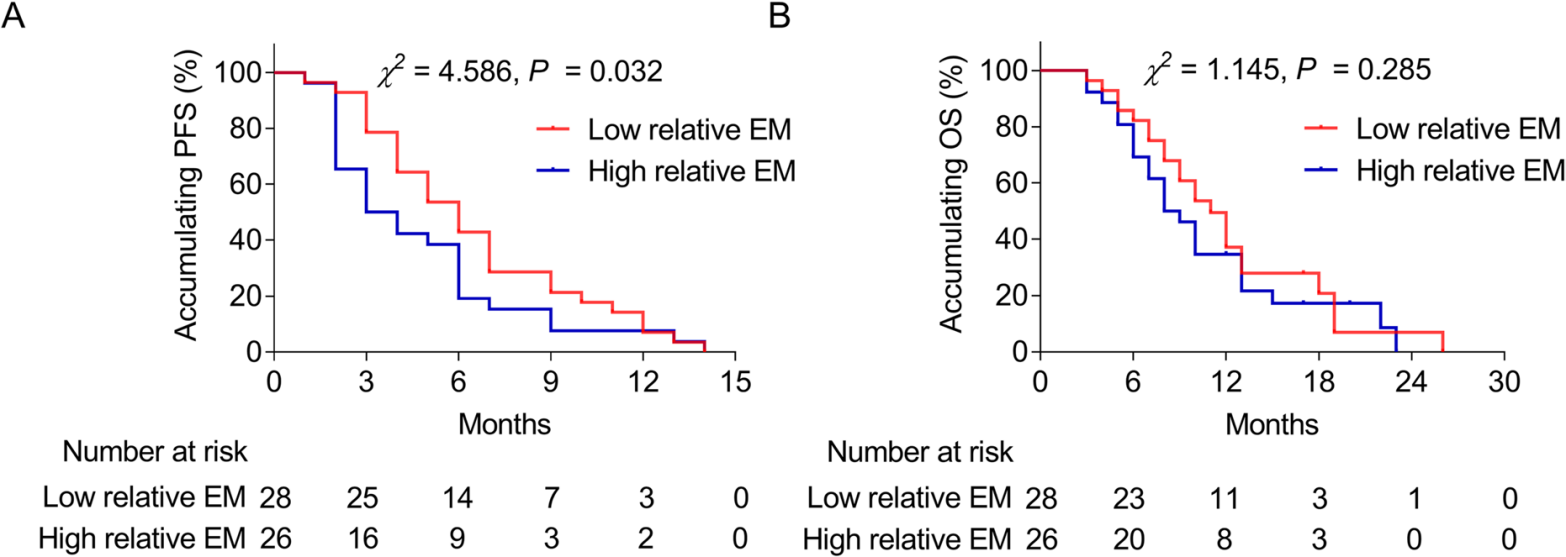
Correlation of relative EM with survival. Association of relative EM with PFS (**A**) and OS (**B**)

**Fig. 2:**
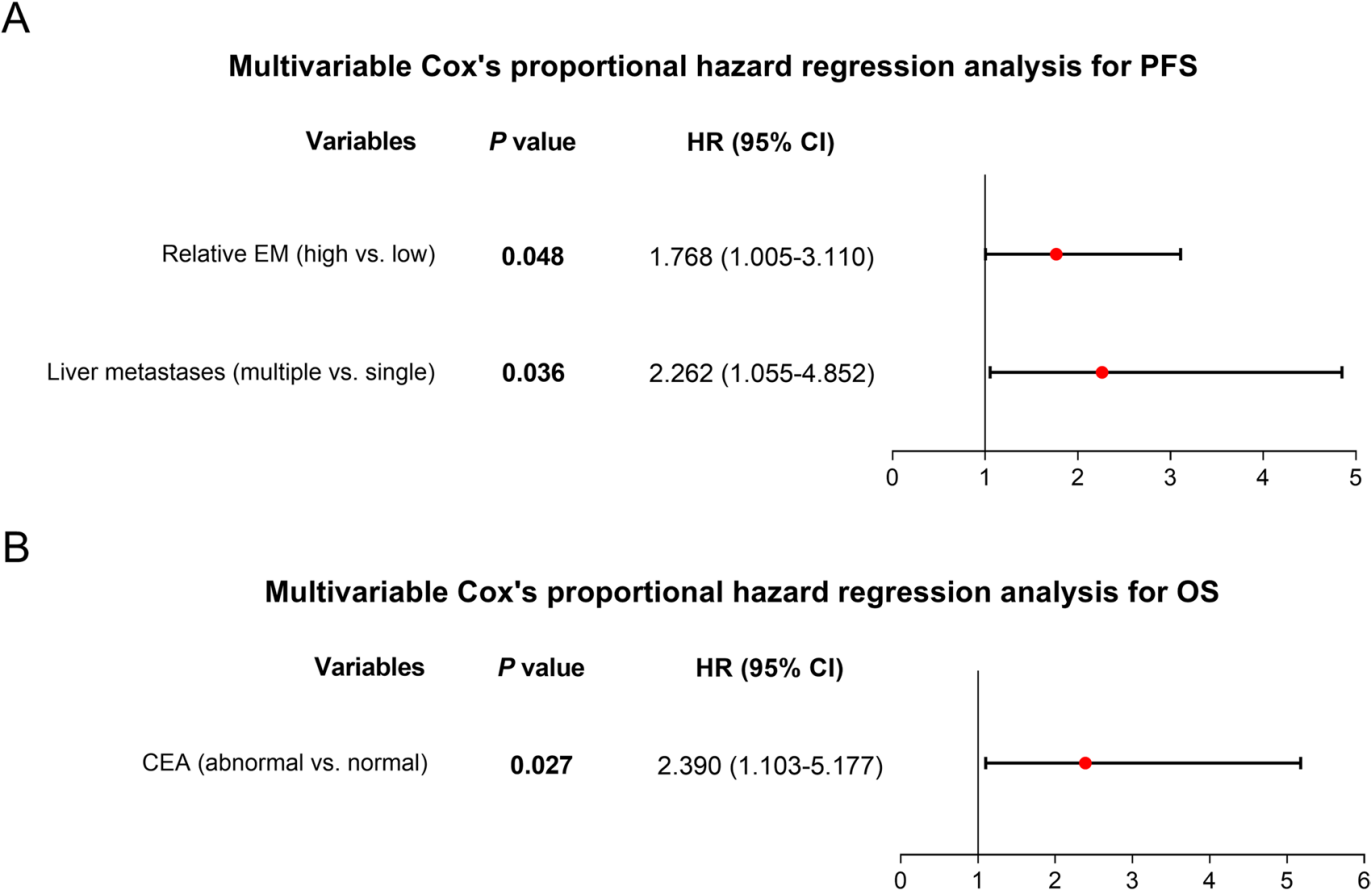
Multivariable Cox proportional hazard regression analysis for survival. Independent factors of PFS (**A**) and OS (**B**)

High relative EM was correlated with decreased PFS (*P* = 0.032) (Fig. 1A). At the same time, no correlation was found between relative EM and OS (*P* = 0.285) (Fig. 1B). In addition, multivariable Cox proportional hazard regression analysis indicated that high relative EM (hazard ratio (HR) = 1.768, *P* = 0.048) and multiple metastases (HR = 2.262, *P* = 0.036) were independently correlated with decreased PFS (Fig. 2A). Moreover, abnormal CEA was independently correlated with decreased OS (HR = 2.390, *P* = 0.027) (Fig. 2B). However, liver metastases (single vs. multiple) and size of liver metastasis were not associated with PFS or OS (Table S1).

## Discussion

Over the decades, evaluation of liver stiffness has been helpful for the management of liver cancer [9–11, 14, 15, 21]. In the past, liver stiffness was mainly evaluated by liver biopsy, while this invasive method might trigger the risk of complications [22, 23]. With the development of medical imaging technology, liver stiffness measured by elastography without invasion is widely applied and is viewed as an indicator of the prognosis of liver cancer. Regarding the correlation of liver stiffness with clinical characteristics in patients with primary liver cancer and liver metastasis, it has been reported that elevated liver stiffness was correlated with a higher tumor stage and fibrosis stage in primary liver cancer [14, 15]. However, the association of liver stiffness with clinical characteristics in pancreatic cancer patients with liver metastasis still needed exploration. In the current study, relative EM was positively associated with liver metastasis ≥ 3 cm among pancreatic cancer patients with liver metastasis. The possible explanations are that (1) the stiffness of liver metastatic lesions was associated with collagen concentration, which could accelerate the proliferation of pancreatic cancer cells at the liver metastatic lesion, consequently leading to elevated liver metastasis size [24, 25], and (2) the stiffness of peripheral liver tissue could affect epithelial-mesenchymal transition and phosphatidylinositol-3-kinase/protein kinase B (PI3K/AKT) signaling in liver metastasis, which consequently promoted the growth of liver metastatic lesions [1, 9, 24, 26]. Combining the above two reasons, plus the fact that relative EM was calculated by the ratio of EM of liver metastatic lesion to EM of peripheral liver tissues, it could be derived that relative EM was positively associated with liver metastasis ≥ 3 cm. Notably, the current study used relative EM value instead of EM value because relative EM value could eliminate the influence of the stiffness of the liver, thus reducing potential deviation.

In terms of the association of liver stiffness with survival in patients with primary liver cancer and liver metastasis, a previous study has reported that elevated liver stiffness was correlated with a decreased 5-year OS, which is also able to predict long-term recurrence among liver cancer patients [27]. Moreover, increased liver stiffness is an independent predictive factor for 1-year recurrence among early liver cancer patients after surgery [15]. Higher liver stiffness is associated with an elevated 3-year mortality rate among liver cancer patients after liver transplantation [28]. A previous study also revealed that reduced liver metastasis stiffness was associated with better treatment response to bevacizumab in patients with metastatic colorectal cancer [29]. However, data on the correlation of liver stiffness with survival in liver metastasis patients are limited. In the current study, elevated relative EM was an independent factor for decreased PFS among pancreatic cancer patients with liver metastasis. The possible explanations are that (1) relative EM was correlated with increased liver metastasis volume (abovementioned), which led to declined PFS among patients; (2) increased relative EM indicated abnormal PI3K/AKT pathway and collagen concentration in liver metastasis, which could not only regulate drug resistance but also modulate antitumor immune microenvironment via enrichment of tumor-associated fibroblasts, consequently affecting PFS among patients [24, 26, 30–32]. Therefore, elevated relative EM was linked with decreased PFS among pancreatic cancer patients with liver metastasis. However, no correlation was found in relative EM with OS among pancreatic cancer patients with liver metastasis. The potential reasons might be that (1) the sample size was relatively small, leading to a low statistical power, and (2) OS could be affected by treatment after disease progression among patients; hence, no correlation was found in relative EM with OS among pancreatic cancer patients with liver metastasis. In the current study, there were five patients with oligo metastasis. These patients all received chemotherapy but not surgical resection of metastasis after considering the potential benefit of surgery and their physical status.

To the best of our knowledge, the current study is the first to explore the clinical role of liver stiffness in pancreatic cancer patients with liver metastasis. Meanwhile, the liver stiffness parameter in the current study was a relative value, which was based on the ratio of EM in liver metastasis to EM in peripheral liver tissue. Thus, relative EM in the current study excluded the potential influence of individual differences on the study findings. However, there were several limitations: (1) the sample size of the current study was relatively small, which could be enlarged in the future; (2) the current study only used one approach to measure EM; thus, more approaches could be applied in future studies; and (3) the clinical value of relative EM in liver metastasis from other cancers, such as colorectal cancer and lung cancer, could be explored.

## Conclusion

In conclusion, high relative EM correlates with unfavorable PFS in pancreatic cancer patients with liver metastasis, whose monitoring may help promote the management of these patients.

### Supplementary Information

**Additional file 1: Fig. S1.** A representative image.

**Additional file 2: Supplementary Table 1.** Correlation of liver metastases with PFS and OS.

## Data Availability

The datasets used and/or analyzed during the current study are available from the corresponding author on reasonable request.

## References

[1] Rahib L, Smith BD, Aizenberg R, Rosenzweig AB, Fleshman JM, Matrisian LM (2014). Projecting cancer incidence and deaths to 2030: the unexpected burden of thyroid, liver, and pancreas cancers in the United States. Cancer Res.

[2] Sung H, Ferlay J, Siegel RL (2021). Global Cancer Statistics 2020: GLOBOCAN estimates of incidence and mortality worldwide for 36 cancers in 185 countries. CA Cancer J Clin.

[3] Wood LD, Canto MI, Jaffee EM, Simeone DM (2022). Pancreatic cancer: pathogenesis, screening, diagnosis, and treatment. Gastroenterology..

[4] Kolbeinsson HM, Chandana S, Wright GP, Chung M (2023). Pancreatic cancer: a review of current treatment and novel therapies. J Invest Surg.

[5] Yamada Y (2022). Present status and perspective of perioperative chemotherapy for patients with resectable pancreatic cancer in Japan. Glob Health Med.

[6] Chen X, Liu F, Xue Q, Weng X, Xu F. Metastatic pancreatic cancer: Mechanisms and detection (Review). Oncol Rep. 2021;46(5):231. 10.3892/or.2021.8182 PMC8444192 34498718

[7] Sohal DP, Mangu PB, Khorana AA (2016). Metastatic pancreatic cancer: American Society of Clinical Oncology Clinical Practice Guideline. J Clin Oncol.

[8] McGuigan A, Kelly P, Turkington RC, Jones C, Coleman HG, McCain RS (2018). Pancreatic cancer: a review of clinical diagnosis, epidemiology, treatment and outcomes. World J Gastroenterol.

[9] Dong Y, Zheng Q, Wang Z (2019). Higher matrix stiffness as an independent initiator triggers epithelial-mesenchymal transition and facilitates HCC metastasis. J Hematol Oncol.

[10] Dulai PS, Singh S, Patel J (2017). Increased risk of mortality by fibrosis stage in nonalcoholic fatty liver disease: systematic review and meta-analysis. Hepatology.

[11] Tatsumi A, Maekawa S, Sato M (2015). Liver stiffness measurement for risk assessment of hepatocellular carcinoma. Hepatol Res.

[12] Zhang CY, Yuan WG, He P, Lei JH, Wang CX (2016). Liver fibrosis and hepatic stellate cells: etiology, pathological hallmarks and therapeutic targets. World J Gastroenterol.

[13] Tacke F, Trautwein C (2015). Mechanisms of liver fibrosis resolution. J Hepatol.

[14] Kim B, Kim SS, Cho SW (2021). Liver stiffness in magnetic resonance elastography is prognostic for sorafenib-treated advanced hepatocellular carcinoma. Eur Radiol.

[15] Wang JH, Li WF, Yong CC, Liu YW, Lu SN, Wang CC (2021). Liver stiffness and insulin resistance in predicting recurrence for early stage hepatoma patients after curative resection. Sci Rep.

[16] Vogl TJ, Martin SS, Johnson AA, Haas Y (2020). Evaluation of MR elastography as a response parameter for transarterial chemoembolization of colorectal liver metastases. Eur Radiol.

[17] Ahmed R, Ye J, Gerber SA, Linehan DC, Doyley MM (2020). Preclinical imaging using single track location shear wave elastography: monitoring the progression of murine pancreatic tumor liver metastasis in vivo. IEEE Trans Med Imaging.

[18] Payen T, Oberstein PE, Saharkhiz N (2020). Harmonic motion imaging of pancreatic tumor stiffness indicates disease state and treatment response. Clin Cancer Res.

[19] Wang K, Lu X, Zhou H (2019). Deep learning Radiomics of shear wave elastography significantly improved diagnostic performance for assessing liver fibrosis in chronic hepatitis B: a prospective multicentre study. Gut.

[20] Dietrich CF, Bamber J, Berzigotti A (2017). EFSUMB guidelines and recommendations on the clinical use of liver ultrasound elastography, update 2017 (long version). Ultraschall Med.

[21] Mak LY, Wong DK, Cheung KS, et al. Role of Serum M2BPGi Levels in Predicting Persistence of Advanced Fibrosis in Chronic Hepatitis B Virus Infection. Dig Dis Sci. 2022;67(11):5127–36. 10.1007/s10620-022-07429-4 35258755

[22] Garcovich M, Faccia M, Di Stasio E (2022). Correlation between QElaXto techniques and supersonic imagine for liver stiffness quantification in chronic liver disease. J Ultrasound Med.

[23] Yoshioka K, Hashimoto S (2012). Can non-invasive assessment of liver fibrosis replace liver biopsy?. Hepatol Res.

[24] Riegler J, Labyed Y, Rosenzweig S (2018). Tumor elastography and its association with collagen and the tumor microenvironment. Clin Cancer Res.

[25] Liu G, Liu R, Shan Y, Sun C (2021). Marine bacterial exopolysaccharide EPS11 inhibits migration and invasion of liver cancer cells by directly targeting collagen I. J Biol Chem.

[26] Dong Y, Xie X, Wang Z (2014). Increasing matrix stiffness upregulates vascular endothelial growth factor expression in hepatocellular carcinoma cells mediated by integrin beta1. Biochem Biophys Res Commun.

[27] Siu-Ting Lau R, Ip P, Lai-Hung Wong G (2022). Liver stiffness measurement predicts short-term and long-term outcomes in patients with hepatocellular carcinoma after curative liver resection. Surgeon.

[28] Amin Fallahzadeh M, Asrani SK, Vahhab E, et al. Prediction of long-term morbidity and mortality after liver transplantation using two-dimensional shear wave elastography compared with liver biopsy. Liver Transpl. 2022;28(10):1618–27. 10.1002/lt.26450 35255183

[29] Shen Y, Wang X, Lu J (2020). Reduction of liver metastasis stiffness improves response to bevacizumab in metastatic colorectal cancer. Cancer Cell..

[30] Li J, Feng D, Gao C (2019). Isoforms S and L of MRPL33 from alternative splicing have isoformspecific roles in the chemoresponse to epirubicin in gastric cancer cells via the PI3K/AKT signaling pathway. Int J Oncol.

[31] Heiserman JP, Nallanthighal S, Gifford CC, et al. Heat shock protein 27, a novel downstream target of collagen type XI alpha 1, synergizes with fatty acid oxidation to confer cisplatin resistance in ovarian cancer cells. Cancers (Basel). 2021;13(19):4855. 10.3390/cancers13194855 PMC8508313 34638339

[32] Mu G, Zhang W, Huang J, Chen Z, Wang J (2022). Research status of tumor-associated fibroblasts regulating immune cells. Zhongguo Fei Ai Za Zhi.

