# Supplementary figures and images for: Elastic modulus-reflected liver lesion stiffness relates to worse prognosis in pancreatic cancer patients with liver metastasis
Source: World J Surg Oncol. 2023 Aug 24;21:262. doi: 10.1186/s12957-023-03140-4 (PMC10463669; doi:10.1186/s12957-023-03140-4)

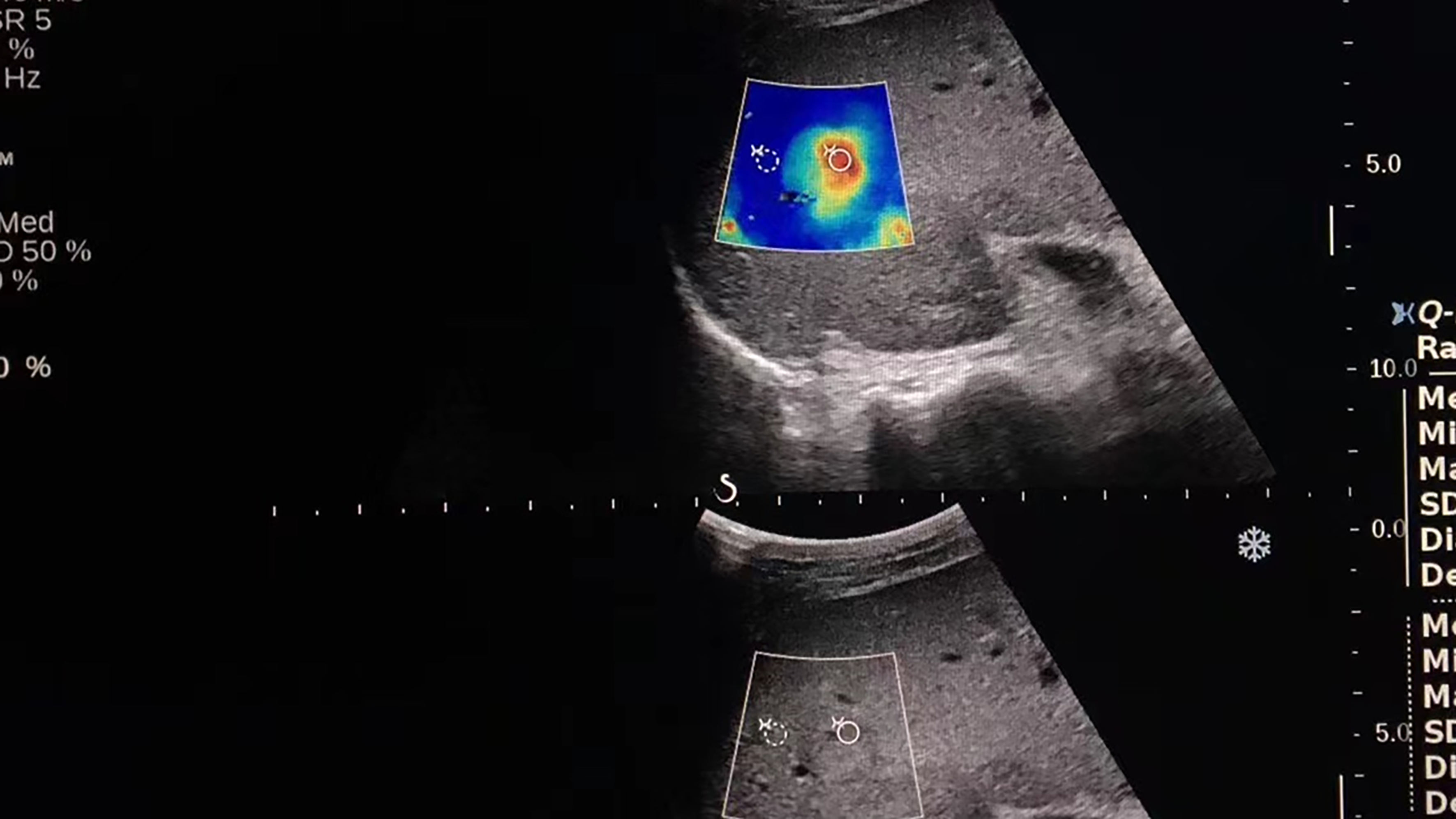

Supplement: Supplementary file 1 — Additional file 1: Fig. S1. A representative image. [file 12957_2023_3140_MOESM1_ESM.tif]
