# Supplementary material for: Elastic modulus-reflected liver lesion stiffness relates to worse prognosis in pancreatic cancer patients with liver metastasis
Source: World J Surg Oncol. 2023 Aug 24;21:262. doi: 10.1186/s12957-023-03140-4 (PMC10463669; doi:10.1186/s12957-023-03140-4)
Supplement: Supplementary file 2 — Additional file 2: Supplementary Table 1. Correlation of liver metastases with PFS and OS. [file 12957_2023_3140_MOESM2_ESM.docx]

**Supplementary Table 1.** Correlation of liver metastases with PFS and OS.

| Items | PFS | | OS | |
| --- | --- | --- | --- | --- |
|  | Median (95%CI), months | *P* value | Median (95%CI), months | *P* value |
| Liver metastases |  | 0.066 |  | 0.060 |
| Single | 6.0 (5.5-6.5) |  | 13.0 (10.6-15.4) |  |
| Multiple | 4.0 (2.9-5.1) |  | 9.0 (7.4-10.6) |  |
| Size of liver metastasis |  | 0.593 |  | 0.094 |
| <3 cm | 5.0 (3.6-6.4) |  | 10.0 (6.8-13.2) |  |
| ≥3 cm | 5.0 (3.5-6.5) |  | 8.0 (5.3-10.7) |  |

PFS, progression-free survival; OS, overall survival; CI, confidence interval.
